# The Book World of Medicine and Science

**Published:** 1904-04-09

**Authors:** 


### The Book World of Medicine and Science.

Memoranda on Infectious Diseases: For the Use of School Teachers. By James W. Allan, M.B., Physician, Glasgow Royal Infirmary. (Bristol: John Wright and Co., 1904. Pp. 23. Price 6d.)

The importance of limiting the spread of infectious disease is now fully recognised, and much has been done in the way of preventive measures. That many zymotic affections are frequently spread by the presence at school of children who are sources of infection to their companions is well known to all. To a certain extent this is unavoidable, as the sufferers in the earlier stages of measles or the later stages of diphtheria may be actively infectious without presenting features readily recognised by an unskilled observer. It is notorious, however, that it is not always these less easily detected cases which are at fault, but that children suffering from well-marked symptoms are also met with. This little book is designed to help the teacher to detect suspicious cases, which can be isolated until examined later by a medical man. Brief notes are given on the more important of the infectious diseases, dealing with their periods of incubation, their more prominent symptoms, and the interval at which school attendance may be safely resumed.

Notes on Alcohol. By Sir Walter Gilbey. (London: Vinton and Co., 9 New Bridge Street. Price 6d.)

This pamphlet contains some interesting information on the production and properties of whisky, brandy, and rum. The author states that the art of separating alcoholic spirit from fermented liquors appears to have been known in the far East from the most remote antiquity. The name "alcohol" indicates that the knowledge of the process spread to Western Europe through the Arabs. Contrary to our own preconceived ideas on the subject, Sir Walter Gilbey holds that so early as the twelfth century the Irish were in the habit of making "usquebagh," a term which is synonymous with "aqua vitæ," and which has since become corrupted into "whisky." In dealing with the special substances whose presence renders properly matured spirits wholesome, the author remarks that spirits of wine are more harmful when taken pure than when taken in the form of good brandy or whisky, so that it may be true, as has been suggested, that whatever these substances may be, they act as antidotes to the action of alcohol. In any case they are of such a subtle nature as to have hitherto eluded analysis, and no device has yet succeeded in hastening the process of maturation of spirits. Altogether, the pamphlet contains a good deal of interesting information on the subject with which it deals.

Ailments of Women and Girls. By Florence Stacpoole, Certificated London Obstetrical Society, Lecturer for the National Health Society, etc. (Bristol: John Wright and Co., 1904. Pp. 238. Price, Cloth, 3s., Boards, 2s. net.)

The writer of this work has evidently taken considerable pains in its compilation, and it is with regret that we must regard much of her industry as misdirected energy. A book is not suited for popular use in which such important subjects as menstrual derangements, cancer, chlorosis and hysteria are discussed in a manner suggestive rather of a medical text-book than a domestic manual. Thus, the writer mentions 13 causes of menorrhagia, and though she states that even a physician cannot determine the cause in any given case without a pelvic examination, such catalogues of symptoms are calculated to alarm the patient without any corresponding benefit. Treatment is dealt with in a somewhat general and sketchy fashion, but though there is a warning against self-drugging in the preface, we find recommended throughout the book a considerable number of prescriptions and proprietary preparations some of them containing active drugs, while the advice to use a lotion of corrosive sublimate (strength not stated) for pruritis vulvæ we must unhesitatingly condemn. It cannot be sufficiently impressed on women suffering from symptons of pelvic disease that they should confide in their medical attendants rather than trust to such guides, which are but too apt to prove blind leaders of the blind.

A Guide to Urine Testing for Nurses and Others. By Mark Robinson, L.R.C.P.&S.Ed. Second edition. (Bristol: John Wright and Co., 1904. Pp. 56. Price 1s. net.)

This is a short and simple account of the physical characters and chemical reactions of the ordinary urinary constituents, with the qualitative tests for the more important abnormal substances which may be present in the urine in disease. No attempt is made to deal with the larger question of their quantitative estimation, except an imperfect account of the use of Esbach's albuminometer, and the statement that the amount of sugar can be calculated by the saccharometer. The book contains sufficient information to enable a nurse to rapidly test a sample of urine and give examples showing how the results of such examination may be reported to the medical attendant.

Dispensing Made Easy: With Numerous Formulæ and Practical Hints to Secure Simplicity, Rapidity, and Economy. By Wm. G. Sutherland, M.B.Aberd. (Bristol: John Wright and Co., 1904. Pp. 102. Price 3s. 6d. net.)

Much discussion has recently taken place as to the propriety of a medical man dispensing his own medicines. Whatever view be taken as to its general desirability, there must always remain cases where it is necessary that this should be done. To the recently qualified man this is frequently that portion of his professional work for which he is least prepared, his training in pharmacy being rarely of a kind that makes for successful dispensing, nor are the larger manuals those from which the help he wants is most readily obtained. With the aid of this little book and the ordinary apparatus it should be possible for anyone to compound the preparations in common use without resorting to any of the more difficult operations in pharmacy. The author lays great stress on economy, and shows how much outlay may be avoided without sacrifice of efficiency. Thus he points out the enormous saving effected by the use of non-alcoholic preparations such as liquid extracts, and the advantages in making up such tinctures from standardised extracts instead of following the processes of the Pharmacopœia. The uses of compressed tablets in place of gargles and for the preparation of douches, injections, etc., and the rapid formation of small active powders from tablet titurates, are dealt with in a practical manner. Numerous excellent formulæ for inexpensive stock preparations, with ample space for manuscript additions, and some practical hints as to dispensing for club and workhouse patients, form a handy volume, which we feel sure will prove useful.

